# Orthanq: transparent and uncertainty-aware haplotype quantification with application in HLA-typing

**DOI:** 10.1186/s12859-024-05832-4

**Published:** 2024-07-16

**Authors:** Hamdiye Uzuner, Annette Paschen, Dirk Schadendorf, Johannes Köster

**Affiliations:** 1 Bioinformatics and Computational Oncology, Institute for Artifical Intelligence in Medicine (IKIM), University Hospital Essen, Faculty of Medicine, University of Duisburg-Essen, Essen, Germany; 2 Department of Dermatology, West German Cancer Center, University Hospital Essen, University Duisburg-Essen, Essen, Germany; 3 German Consortium for Translational Cancer Research (DKTK), Partner Site Essen/Düsseldorf, Essen, Germany

**Keywords:** Haplotype quantification, HLA typing, Bayesian latent variable model, Genomic variants, Uncertainty quantification

## Abstract

**Background:**

Identification of human leukocyte antigen (HLA) types from DNA-sequenced human samples is important in organ transplantation and cancer immunotherapy and remains a challenging task considering sequence homology and extreme polymorphism of HLA genes.

**Results:**

We present Orthanq, a novel statistical model and corresponding application for transparent and uncertainty-aware quantification of haplotypes. We utilize our approach to perform HLA typing while, for the first time, reporting uncertainty of predictions and transparently observing mutations beyond reported HLA types. Using 99 gold standard samples from 1000 Genomes, Illumina Platinum Genomes and Genome In a Bottle projects, we show that Orthanq can provide overall superior accuracy and shorter runtimes than state-of-the-art HLA typers.

**Conclusions:**

Orthanq is the first approach that allows to directly utilize existing pangenome alignments and type all HLA loci. Moreover, it can be generalized for usages beyond HLA typing, e.g. for virus lineage quantification. Orthanq is available under https://orthanq.github.io.

## Background

A haplotype is a biological entity that carries genetic information to be inherited from a single chromosome of same parental origin [1]. It can represent a set of single nucleotide polymorphisms (SNPs) or alleles.

An example for a particularly complex set of haplotypes to quantify is the human leukocyte antigen (HLA) system (the species agnostic name is major histocompatibility complex (MHC)), located in the short arm of chromosome 6, which is a set of genes playing a central role in the immune system [2]. The HLA system consists of three main classes, HLA-I, HLA-II and HLA-III. The first group contains classical loci (i.e. genes) such as HLA-A, HLA-B, HLA-C, nonclassical loci HLA-E, HLA-F, HLA-G and pseudogenes such as HLA-H, the second class the loci DQ (e.g. HLA-DQA1), DR (e.g. HLA-DRB1), DP and the last class consists of genes (e.g. complement system components and tumor necrosis factors (TNFs)) with separate functions involved in immune system [2, 3]. HLA genes show a relatively high genomic variability: the main database for known alleles (IPD-IMGT/HLA) contains currently 37,000 entries^1^ [4]. Following our definition above, each of these alleles can be seen as a haplotype. This variability plays a major role in the activation of specific cells of the adaptive immune system in the context of infection, cancer and autoimmunity.

HLA typing is the process of determining the HLA alleles of each HLA locus/gene of a given sample. In order to simplify characterization and comparison, a nomenclature has been defined that offers unique names for known HLA gene haplotypes [5]. The HLA nomenclature uses a four-field (separated by colons) naming system. The first and second field next to the name of the HLA locus give information about the nucleotide substitution at the protein level. The third field informs about synonymous variants (i.e., not implying a difference at the protein level). The fourth field describes variants in non-coding (i.e., intronic) regions. As an alternative to above HLA nomenclature, the G group notation describes just those sequences of each HLA locus that code for peptide binding domains.^2^ A vast number of alleles are known and HLA loci themselves are highly homologous [6], rendering HLA typing a challenging task.

For determining the HLA type from sequencing data, several approaches have been developed. Optitype [7] adopts an approach where they align reads against exon 2 and exon 3 sequences of HLA-I class alleles and formulates an Integer Linear Program (ILP) that explains the HLA genotype based on the maximum number of mapped reads. HLA-LA [8] relies on linear alignments of reads against the human reference genome plus HLA sequences (including HLA-I and -II) which is then followed by a projection onto a special graph called a Population Reference Graph (PRG) and then resulting in HLA type inference based on a custom optimization process. HLAreporter [9] employs an approach that aligns reads against a comprehensive reference panel (CRP) that consists of HLA alleles, classifies and de novo assembles reads in order to make queries to custom built databases to infer HLA types. HLA-HD [10] firstly prepares a dictionary consisting of exons and introns of all HLA genes. Then, their algorithm works firstly by mapping reads to this dictionary, secondly, read matching to exons and introns that is followed by read weighting and finally, determining the prediction with the highest score. HLAscan [11], similarly, begins with one or two alignment steps and computes a score function which is then followed by inferring HLA types via phasing. arcasHLA [12] counts RNA-sequencing reads with Kallisto [13], constructs a database of the desired IMGT/HLA version and then determines HLA alleles with a genotyping algorithm that takes population-specific allele frequencies into account.

Orthanq differs from these previous approaches in the following ways. First, instead of realigning against the known HLA alleles or phasing variants, it relies on the statistically accurate determination of posterior variant allele frequency (VAF) distributions of the known genomic variation each haplotype (here HLA allele) is made of, while still enabling to use local phasing information (see “Discussion” section for the latter). We show that this can provide a speed advantage without a loss in accuracy. Second, by combining the posterior VAF distributions in a Bayesian latent variable model, we are able to calculate the posterior probability of each possible combination of haplotypes (and their fractions). This allows to report the uncertainty of the predictions, which can be particularly helpful in case of ambiguous possibilities for explaining the data. Moreover, it is desirable in a precision medicine scenario, where knowing about the uncertainty of a prediction can help to decide whether a measurement should be repeated before basing a decision on it. Third, via the choice of the prior distribution the model allows to select between the usual diploid HLA typing and the ability to determine subclonal alleles. The latter can be particularly important in case of tumors, that usually are a heterogeneous mixture of different subclones. Here, HLA typing at subclonal levels can help to predict presented tumor antigens in a more accurate way, thereby potentially improving the success of antigen-targeted immunotherapies. Fourth, while focusing on HLA typing in this first work, the model is generic, such that it can also be used to quantify other kinds of haplotypes. For example, this can be used to quantify virus lineages, as it has been a common task during the SARS-Cov-2 pandemic and still is relevant, for example for wastewater monitoring.

## Methods

We solve the HLA typing problem by generalizing to the problem of quantifying a set of haplotypes that best explains the sequencing reads in a given sample. We first define the general problem and describe our solution. Afterwards we show HLA-typing specific challenges and how to overcome them.

### Haplotype quantification

#### Definitions

*General.* We assume that for the investigated species a reference genome is available that is complete enough to contain all the haplotypes of interest ${\varvec{H}} = \{h_1, h_2, \dots , h_n\}$ (e.g., HLA alleles). Local differences to the reference genome, for example single or multiple nucleotide variants (SNV or MNV), insertions, or deletions (indels) are called variants. Then, each haplotype *h* can be considered a sequence of genomic variants. We define $\{v_1, v_2, \dots , v_k\}$ as the union of all those variants and represent the correspondence of haplotypes and variants as binary matrix $(V_{i,j})_{i=1,2,\dots ,n, j=1,2,\dots ,k}$ with $V_{i,j} = 1$ if haplotype $h_i$ has variant $v_j$ and 0 otherwise. We further define the binary matrix $(C_{i,j})_{i=1,2,\dots ,n, j=1,2,\dots ,k}$ with $C_{i,j} = 1$ if haplotype $h_i$ spans across the locus of variant $v_j$ (but does not have the particular variant) and 0 otherwise.

*Observed variables.* We denote with ${\varvec{Z}} = (Z_1, Z_2, \dots , Z_l)$ the observed DNA fragments in a given sample. For each fragment, we consider a 4-tuple of mapping quality (MAPQ, the posterior probability that the locus the read is aligned to is the wrong one [14]), the alignment of the single read or the read pair (depending on the sequencing protocol), the read sequence(s) and the corresponding base qualities. We further denote for each variant $v_j$ the subset ${\varvec{Z}}_{v_j} \subseteq {\varvec{Z}}$ of fragments that span the variant.

*Latent variables.* We denote with ${\varvec{\Psi }} = (\psi _i \mid \psi _i \in U, i=1,\dots ,n, \sum _{i=1}^n \psi _i = 1)$ the latent fractions of each haplotype in ${\varvec{H}}$ in the given sample. Thereby, *U* denotes the universe of possible fractions, which can for example be continuous ($U = [0,1]$), or discrete (e.g., $U = \{0,0.5,1\}$ for diploid samples). For each variant $v_j$ we further denote with $\theta _j$ the variant allele frequency in the given sample. Finally, we define the same binary latent variables as Köster et al. [15] for each observed DNA fragment $Z_x \in {\varvec{Z}}_{v_j}$. First, we define $\xi _x \in \{0,1\}$ with $\xi _x = 1$ if the fragment is associated with the variant (i.e. has been sampled from the variant allele) and $\xi _x = 0$ otherwise. Second, we define $\omega _x \in \{0,1\}$ with $\omega _x = 1$ if the fragment stems from the locus of interest and $\omega _x = 0$ otherwise.

*Problem.* Our goal is to find approximations of the true haplotype fractions $\hat{{\varvec{\Psi }}}$ that best explain the observed sequencing reads ${\varvec{Z}}$ and to calculate the posterior densities of any provided solutions.

#### Bayesian latent variable model

Under the simplifying assumption that the given haplotypes represent all possible variation of the genome at a locus of interest, the central observation is that the variant allele frequency $\theta _j$ of variant $v_j$ is determined by the fractions of the haplotypes, namely

$$\begin{aligned} \theta _j = \frac{ \sum _{i=1}^n V_{i,j} \psi _i }{ \sum _{i=1}^n C_{i,j} \psi _i }. \end{aligned}$$

In other words, the main evidence for inferring haplotype fractions are the observed variant allele frequencies (i.e., *vertical* observations that are *orthogonal* to the *horizontal* haplotypes that shall be quantified) and their underlying uncertainty. Nevertheless, our approach can easily incorporate phasing information by combining close SNVs into MNVs or SNVs and indels into complex replacements (also see “Generation of candidate haplotypes and variants” section).

In the following, let $Z_x \in {\varvec{Z}}_{v_j}$ be the arbitrary but fixed *x*-th observed fragment spanning variant $v_j$. As defined by Köster et al. [15], for $\omega _x$ we assume

$$\begin{aligned} \omega _x \sim {\text{Bernoulli}}(\pi _x) \end{aligned}$$

with $\pi _x$ being the complement of the probability denoted by the MAPQ value of fragment *x* in the BAM file with the fragment alignments of the sample. While in the single sample case of the original paper $\xi _x \sim {\text{Bernoulli}}(\theta \tau )$ is assumed (with $\tau$ denoting the probability that, if sampled from the variant-affected copy, the fragment indeed covers the variant [15]), we can here directly relate the distribution of $\xi _x$ to the haplotype fractions, that is,

$$\begin{aligned} \xi _x \sim {\text{Bernoulli}} \left( \tau \frac{ \sum _{i=1}^n V_{i,j} \psi _i }{ \sum _{i=1}^n C_{i,j} \psi _i } \right) \end{aligned}$$

Reusing the same modelling as shown by Köster et al. [15], this leads to the likelihood function

$$\begin{aligned} \Pr ({\varvec{Z}}_{v_j} \mid \psi _1, \psi _2, \dots , \psi _n) = \prod _{Z \in {\varvec{Z}}_{v_j}} \Pr \left( Z \mid \theta _j = \frac{ \sum _{i=1}^n V_{i,j} \psi _i }{ \sum _{i=1}^n C_{i,j} \psi _i } \right) . \end{aligned}$$

In practice, this means that the likelihood function can be directly obtained by applying Varlociraptor to the data (see “Utilizing varlociraptor to obtain variant allele frequency distributions” section). Consequently, the likelihood of haplotype fractions given all variants ${\varvec{V}}$ is

$$\begin{aligned} \Pr ({\varvec{Z}} \mid \psi _1, \psi _2, \dots , \psi _n) = \prod _{j=1}^k \Pr ({\varvec{Z}}_{v_j} \mid {\varvec{\Psi }}). \end{aligned}$$

Using Bayes theorem, the posterior is thus

$$\begin{aligned} \Pr ({\varvec{\Psi }} \mid {\varvec{Z}}) = \frac{\Pr (\psi _1, \psi _2, \dots , \psi _n)\Pr ({\varvec{Z}} \mid \psi _1, \psi _2, \dots , \psi _n)}{\Pr ({\varvec{Z}})}. \end{aligned}$$

The marginal probability $\Pr ({\varvec{Z}})$ depends on the fraction universe. In the continuous case ($U = [0,1]$), it is

$$\begin{aligned} \int _0^1 \int _0^1... \int _0^1 {\mathbbm {1}_{\sum _{i=1}^n} \psi _i = 1} \Pr (\psi _1, \psi _2, \dots , \psi _n)\Pr ({\varvec{Z}} \mid \psi _1, \psi _2, \dots , \psi _n) d\psi _1 d\psi _2 \dots d\psi _n \end{aligned}$$

with $\mathbbm {1}_{\sum _{i=1}^n \psi _i = 1}$ being an indicator function that ensures that the sum of the chosen haplotype fractions does not exceed 1. In the discrete case (e.g., $U = \{0, 0.5, 1\}$), it is

$$\begin{aligned} \sum _{\psi _1, \psi _2, \dots , \psi _n \mid \psi _i \in U, i=1,\dots ,n, \sum _{i=1}^n \psi _i = 1} \Pr (\psi _1, \psi _2, \dots , \psi _n) \Pr ({\varvec{Z}} \mid \psi _1, \psi _2, \dots , \psi _n). \end{aligned}$$

By choosing the reference genome, the haplotype set, the prior ($\Pr (\psi _1, \psi _2, \dots , \psi _n)$), and the universe, the model can be configured for various kinds of scenarios. In this manuscript, we will focus on HLA typing, but different universes and priors can for example be used for virus lineage quantification.

#### Practical considerations for model evaluation

As can be easily seen, calculating the marginal probability is computationally expensive. In the continuous case, it would require $\mathcal {O}(q^n)$ with *q* being the number of grid points in the integral (e.g., using quadrature integration) and *n* being the number of considered haplotypes. To speed up the calculation, one can utilize Markov chain Monte Carlo methods (which we want to explore in the future). Further, it is possible to heuristically discard haplotypes that obviously do not reflect the observed VAFs at all upfront. This can be done using linear optimization.

First, we consider for each variant $v_j$ the maximum a posteriori allele frequency $\hat{\theta }_j$ as it is reported by Varlociraptor [15]. We restrict the set of considered variant indices to $W \subseteq \{1, 2, \dots , k\}$ such that each variant $v_j$ with $j \in W$ is covered by all the considered haplotypes. This is necessary to avoid the normalization factor when calculating the haplotype fraction induced allele frequency (see “Bayesian latent variable model” section) in the linear program. We minimize

$$\begin{aligned} \sum _{i=1}^n \left| \sum _W \psi _i V_{i,j}-\hat{\theta }_i \right| \end{aligned}$$

subject to

$$\begin{aligned} & \psi _{i} \in [0,1],i = 1,2, \ldots ,n \\ & \sum\limits_{{i = 1}}^{n} {\psi _{i} } = 1 \\ \end{aligned}$$

The optimization function minimizes the distance between the haplotype fraction induced allele frequency and the observed maximum a posteriori estimate of the allele frequency of each variant. The constraints ensure that the chosen fractions sum up to 1. By keeping only haplotypes with $\psi _i \ge t$ and extending this set with haplotypes that have either the same set of variants or at most *m* more or less (with both *t* and *m* being command line parameters of Orthanq), we can obtain a set of haplotypes that is drastically reduced to exclude those that are unlikely to play a role in favorable solutions. By grouping those haplotypes into equivalence classes of haplotypes that are sufficiently similar to become de facto mutually exclusive in any solution and evaluating combinations of those classes instead of combinations of all LP-selected haplotypes, the search space for the statistical model can be further reduced (we will explore this in the future).

#### Utilizing Varlociraptor to obtain variant allele frequency distributions

As outlined above, the likelihood $\Pr \left( Z \mid \theta _j = \frac{ \sum _{i=1}^n V_{i,j} \psi _i }{ \sum _{i=1}^n C_{i,j} \psi _i } \right)$ can be obtained directly from Varlociraptor. To this end, Varlociraptor offers the ability to specify so-called variant calling scenarios, which allow to configure all relevant aspects of the underlying latent variable model. The scenario consists of a definition of species specific prior assumptions (like the expected heterozygosity), the definition of samples, and the definition of events of interest, all specified in a YAML^3^ based domain specific language. The specifications depend on the use case and can be customized in any way.^4^ In order to simplify the usage, Orthanq offers a subcommand that hides the scenario specification details and applies reasonable defaults. Specifically, we define a single sample, set the universe to be continuous and uniformly distributed and define a simple event that just checks for the presence of a variant.

### HLA-typing specific considerations

**Fig. 1:**
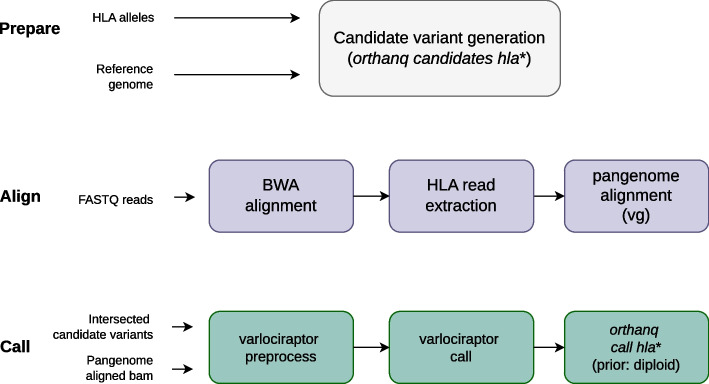
General workflow of HLA typing with Orthanq. The workflow consists of three main parts. “Prepare” consisting of candidate variant generation, “Align” containing the two-step alignment process that each sample undergoes, “Call” including both Varlociraptor for calling variants and Orthanq for calling haplotypes with the latter resulting in type allele prediction, with diploid priors to be used with healthy samples (steps with asterisk (*) are performed by respective Orthanq subcommands)

In the following, we show how the Orthanq model can be used to perform HLA typing. Figure 1 provides an outline of the involved steps. We first generate candidate variants from comparing known HLA alleles against the reference genome. Second, we have to align reads against the reference genome, taking particular care of the homology induced challenges occurring at HLA loci. This step can obviously be shared with other analyses, e.g. for variant calling. Finally, the candidate variants are preprocessed and called with Varlociraptor, providing the required input for HLA typing with Orthanq.

#### Generation of candidate haplotypes and variants

Orthanq expects candidate haplotypes and variants to be given in BCF/VCF format. It provides a subcommand for automatically generating them from a given FASTA file with haplotype sequences and a common reference genome for the species. The subcommand uses minimap2 [16] to align each haplotype sequence against the reference genome and infers SNVs, MNVs and indels from the alignments, representing them in a VCF with one column per haplotype. Thereby, the column fields represent the entries of the matrices *V* (GT field) and *C* (C field) (see “Definitions” section). As mentioned above, while the central evidence used for haplotype fraction inference are variant allele frequencies, candidate variants can easily be grouped into locally phased representations like MNVs or complex replacements since Varlociraptor can handle all of these as well.

For the evaluation of HLA typing presented in this paper, we used all known HLA alleles from the IPD-IMGT/HLA sequence database v3.32, excluding unconfirmed alleles and those with a population allele frequency $< 0.05$ according to the Allele Frequency Net Database [17, 18] as input haplotype sequences and GRCh38 version 106 from Ensembl as reference genome. For now, we did not group individual variants into MNVs or complex replacements (but aim to pursue this in future work, see “Definitions” section). We run Orthanq independently for each HLA locus. Therefore, the candidate matrix was intersected with HLA genes.

#### Pangenome based two-step alignment strategy

We adopt a two-step alignment strategy to ensure that reads align to the reference genome in the most accurate and efficient way. Naturally, the genetic variation of the investigated individual is not represented in a linear genome. The read mapper therefore has to decide about the optimal placement of a read solely based on the linear reference genome. This can lead to the situation that a variation in the individual’s genome generates a homology to a different location of the linear reference genome, pulling the read to a wrong place. Subsequently, the misplaced reads can lead to false positive or false negative variants, and thereby wrongly inferred HLA types. To overcome this issue, we utilize a graph based pangenome read alignment strategy. In graph based reference pangenomes, differential paths represent the known variants. If the pangenome is built to contain most of the relevant genetic variation of a species, wrong read placements can be reduced substantially, and more accurate mapping qualities can be reported [19] Since, at least on some hardware, pangenome read alignment can still be slower than linear reference genome alignment and we only require the maximum alignment accuracy for reads coming from HLA genes, we conduct a two-step approach. Reads are first aligned to the linear genome (GRCh38.p13) by bwa mem with default parameters. Second, we extract reads that map to any HLA class I and II locus based on their genomic coordinates. While HLA class III loci are technically possible as well, the IPD-IMGT/HLA database currently does not hold corresponding allele sequences. The extracted reads are then aligned to a human pangenome graph that captures the most relevant genetic variation [19] using vg giraffe [20, 21].

## Results

### Evaluation

In order to compare our novel model implemented in Orthanq with state of the art approaches for HLA typing, we evaluated in total 99 samples for which each sample’s HLA-A, HLA-B, HLA-C, and HLA-DQB1 alleles have been determined and validated by a clinical laboratory Chin et al. [22] and either computationally or experimentally determined by Abi-Rached et al. [23]. The latter is offered by The International Genome Sample Resource (IGSR) as of May 2024. Those entail (a) whole exome sequencing (WES) samples (all samples from the population of CEU, if there is no discrepancy in the ground truth) from 1000 Genomes Project [24] that are listed under the project number PRJNA59853 on SRA,^5^ (b) 3 whole genome sequencing (WGS) samples from Illumina platinum genomes [25] that are listed under the project number PRJEB3381 on SRA,^6^ and (c) the WES sample HG002 (SRA ID SRR2962669) of the NA12275 individual from the Genome In a Bottle project (GIAB) [26] under the project number PRJNA200694 on SRA.^7^ We provide a Snakemake report with all analysis results together with the used code, parameters, and software versions via Zenodo.^8^ In the subsequent analysis, the GIAB sample (sample name is SRR2962669 and alias is “D1_S1_L001” in the report) is evaluated separately, because its HLA types are given in G group notation in the benchmark [22]. A summary table across all samples can be found in the Snakemake report^9^ (section “sample sheet”).

*Comparison with state-of-the-art HLA typers* For comparison, we chose arcasHLA (v0.5.0), Optitype (v1.3.5), and HLA-LA (v1.0.3), because these tools have relatively recent releases and appear to be continuously maintained. For the fairest evaluation, IPD-IMGT/HLA release version 3.32 was preferred for Orthanq and arcasHLA since that is the version that HLA-LA uses in its predictions. For Optitype, the database version cannot be customized. All tools were executed with their default parameters. For Orthanq we configured a diploid prior, in other words, we assumed that all evaluated samples are diploid at their HLA loci.

### Accuracy and call rate

On the evaluated samples, we calculate accuracy and call rate across all samples excluding HG002 sample. The latter is evaluated separately, because the true alleles are given as G groups (see “Background” section) only. We define the call rate as the fraction of samples for which the evaluated tool provides a prediction among all loci and samples. We define the accuracy, as the fraction of correctly predicted HLA genotypes across all loci and all called samples (i.e., excluding samples where the tool did not provide a prediction). The genotype of an HLA locus is thereby defined as a pair of HLA alleles defined by the first two fields of the nomenclature, in other words, only considering non-synonymous coding variants, to be compliant with the provided benchmark dataset by Abi-Rached et al. [23]. In fact, Orthanq is capable of reporting three fields of the nomenclature as well. For two loci of one individual (NA12874, locus A &B), Abi-Rached et al. [23] report multiple HLA genotypes. In this case, any of the listed alleles are accepted as accurate for the tool.

HLA-LA and Orthanq offer multiple predictions per locus. Orthanq may output several combinations of alleles with the same density, while HLA-LA may output several different alleles per chromosome for one locus. Then, the sample is accounted as accurate if one of the combinations (for Orthanq) and alleles (for HLA-LA) matches the ground truth for the corresponding sample.

**Fig. 2:**
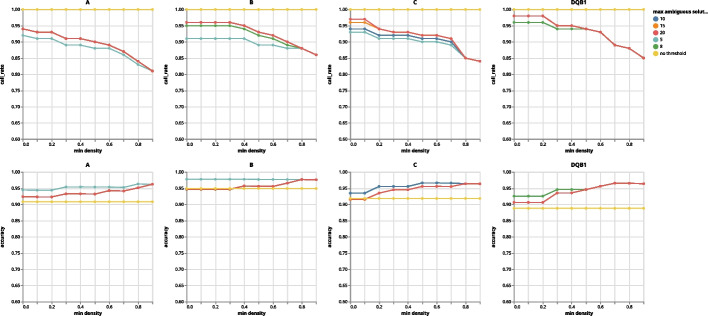
Call rate and accuracy depending on thresholds used to determine a considered call in the performed benchmark, stratified by HLA locus. Horizontal axes show the minimum required density, colors depict maximum allowed haplotype solutions with same maximum density

Since Orthanq reports a posterior probability for each provided solution as a result of uncertainty quantification, it can be that some solutions have identical posterior probabilities, yet suggesting different HLA types. In order to show the tradeoff between the threshold and accuracy and call rate, we varied threshold values to obtain the effect on accuracy and call rate as shown in Fig. 2. We varied the first threshold from 5 to 10 (and omitting it entirely - no threshold) and the second from 0.0 to 0.9. It can be seen from the plot that that as expected, choosing more permissive thresholds increases the call rate at the expense of loosing some accuracy.

Therefore, it is up to the user to choose a cutoff for considering a prediction to be made, for example by controlling the local false discovery rate. Here we kept a prediction if Orthanq reported $\le 5$ solutions of equal probability which summed up to $> 0.7$.

**Fig. 3:**
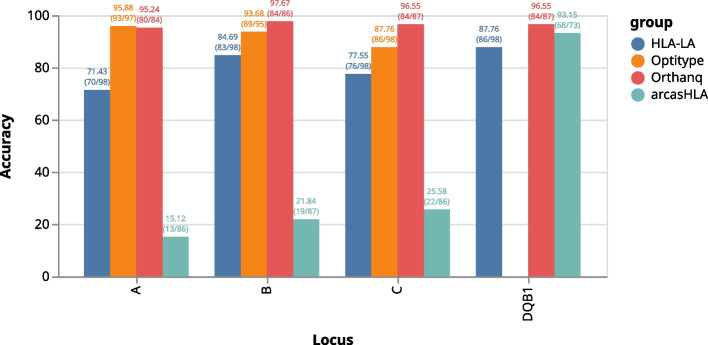
Accuracy of HLA-LA, Optitype, arcasHLA and Orthanq for 98 samples (excluding GIAB). For code, software, and parameters, see section “Accuracy” in the Snakemake report

**Fig. 4:**
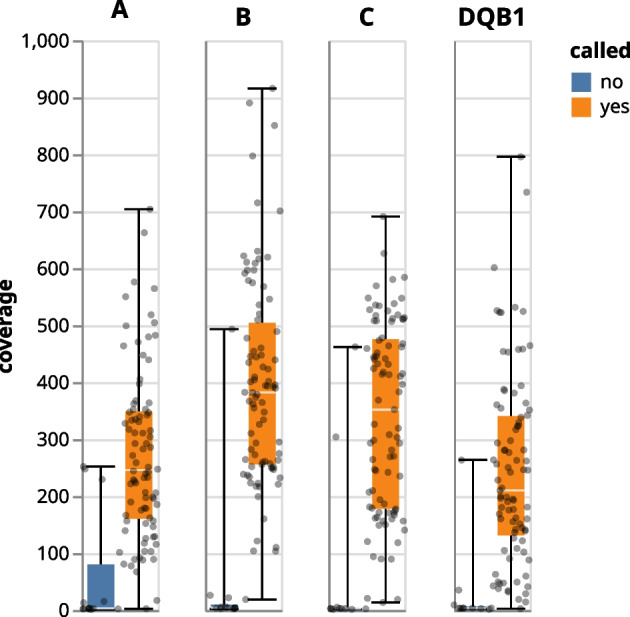
Boxplot of coverages for samples belonging to 1000 Genomes, a trio from Illumina Platinum Genomes (N = 98). For code, parameters, and software versions, as well as detailed access to the underlying data, see the Snakemake report

As can be seen in Fig. 3, Orthanq provides the best accuracy over all loci, followed by Optitype, which is however unable to predict alleles for HLA-DQB1. On the HLA-A locus, Optitype’s accuracy slightly better, though one could as well argue that this difference so marginal that both tools have about the same accuracy on HLA-A. After Orthanq and Optitype, HLA-LA comes next which is then followed by arcasHLA. Given our filtering approach (see above paragraph), call rates of Orthanq are lower than those of Optitype. However, this does not mean that Orthanq would not provide a call in such cases, it just offers various alternatives and weak posterior probabilities, thereby reporting the increased uncertainty in the data. The benefit of this is nicely illustrated in the lower accuracy of Optitype on non HLA-A loci that manifests itself predominantly on samples with a lower coverage (see Fig. 4 for sample coverages). For the GIAB sample, Orthanq and HLA-LA predict the correct HLA alleles for all loci. Optitype predicts the correct HLA alleles for all loci except HLA-DQB1, as it does not provide predictions for this locus. ArcasHLA only predicts HLA-DQB1 for this sample, for which it however reports the correct alleles. G notations of HLA predictions are given in the following: A*01:01:01/A*26:01:01 for A*01:01:01G/A*26:01:01G, B*35:08:01/B*38:01:01 for B*35:08:01G/B*38:01:01G, C*04:01:01/C*12:03:01 for C*04:01:01G/C*12:03:01G, DQA1*01:05:01/DQA1*03:01:01 for DQA1*01:01:01G/DQA1*03:01:01G, DQB1*03:02:01/DQB1*05:01:01 for DQB1*03:02:01 G/DQB1*05:01:01 G. In the Snakemake report 10 (section “Evaluation (all tools)”), a table that shows the predicted HLA alleles for each sample across all tools can be found.

**Fig. 5:**
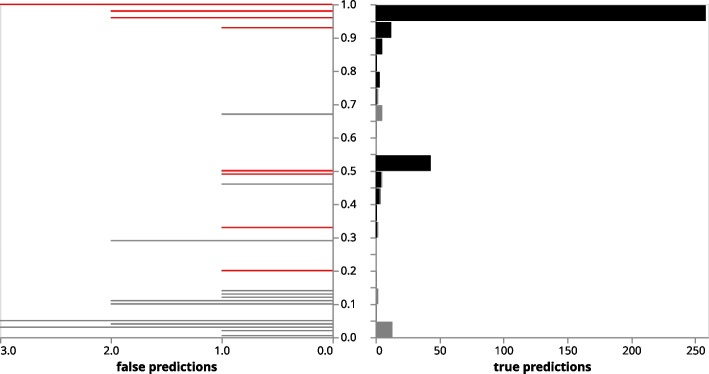
Density distributions of True Positive (black), False Positive (red) and uncalled predictions (gray) for 98 samples (excluding GIAB). Uncalled predictions are split into “true & uncalled” (right) and “false & uncalled” (left) if the predictions do not meet the threshold criteria. For code, software, and parameters, see section “Orthanq density accuracy” in the Snakemake report

With Orthanq, we aim to not only correctly quantify given haplotypes (or in this case correctly predict HLA alleles), but also aim to accurately report the uncertainty of the prediction. While with the given benchmark data the numbers of false predictions are too small to validate the provided posterior probabilities numerically, we can still check whether the tendency of observing a false prediction is increasing with decreasing posterior probabilities. Figure 5 shows the count of records for correct (right) and false (left) predictions compared to the posterior probabilities reported by Orthanq across all evaluated samples. The analysis considers predictions that pass the thresholds true positives or false positives and uncalled if they don’t. The latter ones are predictions that do not meet the threshold criteria, but would have been true positive or false positive if they did. The aforementioned predictions are split into two, “uncalled” true predictions are found on the right, whereas “uncalled” false predictions are found on the left side of the plot. From Fig. 5, it can be seen that more than half of the uncalled predictions (27/48) still contain the true combination of alleles, just ambiguously together with other possible solutions (for data points underlying, see “all predictions density table” in the section “Orthanq density accuracy” in the Snakemake report). The density plots, solution plots generated by Orthanq for each sample automatically, can all be found in the Snakemake report. In addition, it can be seen that false predictions occur in higher fractions at low posterior probabilities.

### Runtime performance and memory consumption

**Fig. 6:**
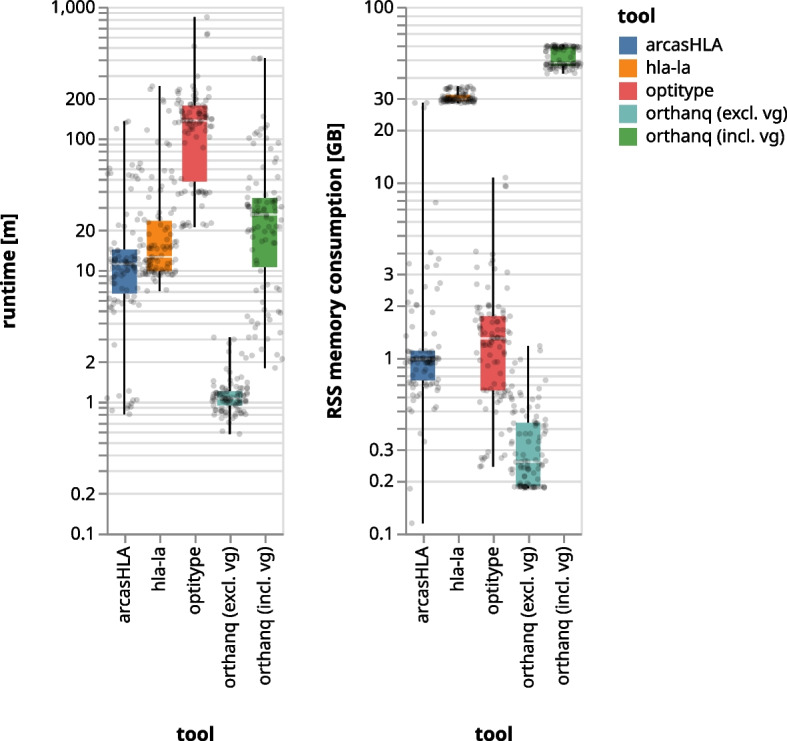
Boxplot of runtimes of Orthanq, Optitype, HLA-LA and arcasHLA for samples belonging to 1000 Genomes, a trio from Illumina Platinum Genomes and the GIAB sample (N = 99). For code, parameters, and software versions, as well as detailed access to the underlying data, see “Runtime performance” section in the Snakemake report

We also compare the performance of Orthanq against the other tools in terms of runtime and memory consumption. Experiments were conducted on a compute server (Intel Xeon Silver 4216, 2.1 GHz, 188 GB RAM), providing 40 CPU cores to each tool. Most tools contain several steps that have to be executed for each considered sample (e.g., preprocessing of read alignments and candidate variants with Orthanq, followed by the actual haplotype quantification). We measured the per sample runtime as the sum of all of these steps and the per sample memory usage as the maximum across all steps. We did not include ordinary linear genome read alignment or usual common preprocessing steps like PCR deduplication in the measurements, as we expect this to happen anyway, independently of HLA typing (e.g. for variant calling). Figure 6 shows the results for all evaluated samples. When excluding vg-related steps, Orthanq is the fastest across both preprocessing and calling and has the least memory usage (see “Discussion” section). If vg is included, Orthanqs runtimes are still faster than Optitype (the overall second best performing in terms of accuracy), while being higher than HLA-LA and arcasHLA and having the highest memory usage of all tools during the vg step. However, pangenome alignment with vg specifically for HLA typing will become unnecessary in the near future (see “Discussion” section).

### Reporting uncertainty

**Fig. 7:**
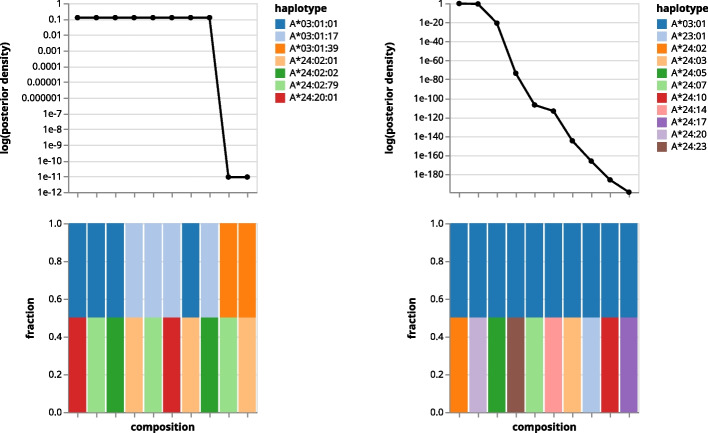
Posterior densities of 10 solutions for 1000 Genomes sample SRR702070, using 3-field (left) and 2-field (right) HLA nomenclature. The shared x axis shows the different solutions and the y axes show the corresponding log-scaled posterior densities and color-coded fractions attributed to each allele. The HLA truth for this sample is given in the following: A*03:01/A*24:02, B*07:02/B*39:06, C*07:02/C*07:02, DQB1*06:02/DQB1*06:02. For code, parameters, and software versions, as well as analogous plots across all evaluated samples, see “Orthanq detailed solutions” section in the Snakemake report

For each locus, Orthanq reports multiple possible combinations of haplotype fractions (here HLA alleles). Each solution is reported with its posterior densities (or probabilities if the universe is discrete, like it is the case here, where diploid genomes are assumed). For visual assessment, Orthanq offers the possibility to plot the first n solutions, with n being configurable. In Fig. 7, the first ten solutions sorted by their log-scaled posterior densities are given. For the given sample, there are eight events that share the same densities which can easily be deduced from line plot. The bar plot shows the corresponding fractions of solutions, 0.5 for each allele, which is to be expected in a “diploid” healthy sample. Since the third field of HLA nomenclature represents synonymous mutations, it is less relevant in practice. Therefore, Orthanq offers its omission (Fig. 7 right part), summing densities of solutions with the same two-field alleles, and it provides both three-field and two-field resolution outputs.

**Fig. 8:**
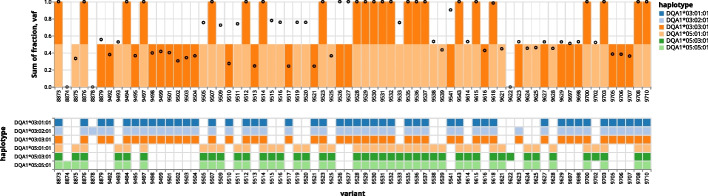
Bar plot transparently displaying the model decision in relation to Varlociraptor’s maximum a posteriori allele frequency estimates for each variant. The x axis shows variant IDs and y axis shows variant allele frequencies (black circles) as well as the sum of haplotype fractions (as stacked bar plot). Thereby, the sum of the fractions of haplotypes having a certain variant should match the observed variant allele frequency. Each color represents an HLA allele. For this sample Orthanq predicts DQA1*05:01:01 (light orange) and DQA1*03:03:01 (dark orange). The matrix plot below the bar plot shows for each evaluated haplotype which variants it hosts. For code, parameters, and software versions, as well as analogous plots across all evaluated samples, see “Orthanq detailed solutions” section in the Snakemake report

For even deeper investigation of Orthanqs predictions, the tool allows to assess the contributions of individual variants to the final haplotype prediction. In Fig. 8, the contribution of allele frequencies coming from variants can be checked to see how they affect the linear program and the statistical model decision.

On average, across all benchmark samples, the LP reduces the number of candidate haplotypes to be considered by 75%. A histogram of pruned haplotypes can be found in the Snakemake report in the supplement.

### Pangenome based avoidance of alignment artifacts

As mentioned before (“Pangenome based two-step alignment strategy” section), we utilize a two-step alignment strategy: the usual linear reference genome alignment, followed by pangenome based realignment of reads that originate from HLA loci. This is needed because the high homology of HLA loci can give rise to incorrect read placements caused by the variants occurring in the investigated individual. Additional file 1 shows an example that compares alignments with linear and pangenome approach. With linear reference genome alignment (using bwa mem), the variant 6:29943462:T>C is called, which does not belong to the known homozygous HLA type A*02:01 of this sample. Using a pangenome reference with vg giraffe [20, 21] though causes the reads supporting the variant to get a mapping quality of zero, denoting that they cannot be uniquely placed to this location and an alternative placement is equally possible. In consequence, the variant is not called by Varlociraptor, and therefore does not misguide the HLA typing towards a wrong allele. Currently, Orthanq does not offer an approach without including pangenome alignment.

## Discussion

Using previous publications, we curated a comprehensive and cleaned up set of 99 benchmark samples with known and validated HLA types. Apart from its additional benefits in terms of the provided uncertainty estimation and the ability to closely investigate the involved variants, Orthanq overall manages to outperform other HLA typers in terms of accuracy on the investigated samples (with the exception of HLA-A, where Orthanq’s accuracy is about the same as that of Optitype). Results show that Orthanq tends to be more conservative than other HLA typers: instead of always providing one solution, it sometimes lists multiple solutions with the same (weaker) posterior probability, thereby reflecting uncertainty in the data. It can be seen that such cases are in general reflecting weak evidence (and correspondingly a higher chance for wrong predictions if uncertainty would not be estimated) like low read coverage in the respective sample.

Under the assumption that pangenome based read alignment is the upcoming standard approach (as e.g. illustrated by the fact that it is nowadays the default for the Illumina Dragen pipeline), Orthanq also provides the by far most favorable runtime and memory requirements: it can directly work from pangenome aligned reads with the accuracy reported in this paper given that a sufficiently representative pangenome was used as e.g. presented by Liao et al. [19]. Moreover, as Orthanq relies on pangenome alignments, any performance based improvement in pangenome aligners will directly affect runtime and memory performance of Orthanq. When including measurements for pangenome based read alignment (vg), Orthanq still provides a runtime comparable to the overall second best HLA typer, Optitype.

Orthanq obtains its evidence from variants that are retrieved by aligning given haplotypes or alleles to a reference genome. Currently, the variants are encoded as SNVs and small indels. In the future, we strive to group variants that are close to each other into small haplotypes, represented as complex replacements and multiple nucleotide variants (MNVs). This way, the Varlociraptor based calling utilized by Orthanq will automatically deliver evidence from reads spanning multiple variants, which should further improve the already very high accuracy provided by Orthanq. Moreover, we also expect this to further improve the reported certainty of calls (and thereby Orthanq’s call rate in the context of the evaluation in this manuscript), as formerly ambiguous solutions will sometimes become more distinguishable.

Since the model is agnostic of the considered universe of possible haplotype fractions (see “Definitions” section), it can also be applied to predict subclonal HLA types of for example tumor samples. While such subclonal HLA typing requires further optimizations in the implementation (see “Practical considerations for model evaluation” section), it could provide important information for designing tumor-antigen targeted immunotherapies, since this would allow to detect the evolution of tumor subclones which no longer present specific tumor antigens due to HLA allele loss or to predict subclone specific tumor antigen binding affinities.

Orthanqs model evaluation is in principle exponential in the number of considered haplotypes. However, using linear programming, the number of haplotypes to consider can be dramatically reduced, enabling practical feasibility when constraining the universe of possible haplotype fractions (see LP pruned haplotypes in the supplementary Snakemake report). In the future, we plan to further improve the speed by exploring an equivalence class based approach preprocessing de facto mutually exclusive solutions, as well as statistical approximations (see “Practical considerations for model evaluation” section).

By utilizing variant based evidence generated by Varlociraptor, Orthanq can also make direct use of Varlociraptors advanced capabilities for defining relations between samples, e.g. in terms for inheritance (mendelian and clonal) and contamination. This way, for the first time, contamination or pedigree information and complex sample scenarios like longitudinal studies can be taken into account on a statistical level for HLA typing.

While Orthanq’s model is capable to type any kind of HLA locus, in practice, Orthanq is currently able to type HLA-A, HLA-B and HLA-C from class I genes and HLA-DQA1 and HLA-DQB1 from class II genes. The reason for this practical limitation is that HLA-DRB1 (the second class II gene) requires a refinement of the way we determine candidate variants that can deal with the high sequence similarity between DRB1 and DRB5 (an issue that is also known to another HLA typer, HLA-reporter [9]). Once this has been implemented, we will extend our benchmarking towards all class II genes.

Finally, Orthanq has applications beyond the typing of HLA alleles, because it can handle any kind of haplotype and reference genome. For example, we plan to apply Orthanq to quantify virus lineages in patient or wastewater samples, e.g. for monitoring SARS-Cov-2, influenza or other future virus outbreaks, while, for the first time, being able to accurately report the involved uncertainties and pinpoint individual mutations in addition to the general lineages.

## Conclusion

With Orthanq, we presented a novel statistical approach for the quantification of haplotypes, that, for the first time, allows to statistically report the uncertainty of the predicted quantifications. We showed how Orthanq excels in performing HLA typing, which is particularly challenging because of the high variation and homology between HLA loci. Orthanq not only predicts the HLA types of samples, it moreover offers detailed insights into the associated variants and their contribution to the model decision and the estimated posterior probabilities (or densities). Moreover, this offers the ability to investigate the HLA loci of a sample beyond the known HLA alleles (but still within their context) and assess the potential impact of additional (e.g. subclonal) variation. Finally, Orthanq works out-of-the-box with any combination of HLA loci, database versions, and reference genomes, as it does not rely on any specialized prebuilt indexes.

## Availability

Orthanq is implemented as an open-source, MIT licensed software based on the Rust programming language. It can be reached under https://orthanq.github.io and installed via Bioconda [27]. All the plots generated in this paper, along with their underlying tabular data are provided together with the used code and parameters in the supplementary Snakemake report on Zenodo.^11^ Moreover, the entire codebase of the performed evaluation can be found under https://github.com/orthanq/orthanq-evaluation and on Zenodo.^12^

## Data Availability

The datasets generated during and/or analysed during the current study are available in the EMBL-EBI European Nucleotide Archive repository (https://www.ebi.ac.uk/ena/ with project IDs: PRJNA59853, PRJEB3381 and PRJNA200694]) [24–26].

## Footnotes

1 October 2023.

2 https://hla.alleles.org/alleles/g_groups.html.

3 https://yaml.org/.

4 https://varlociraptor.github.io/docs/calling/#generic-variant-calling.

5 https://www.ncbi.nlm.nih.gov/bioproject/PRJNA59853.

6 https://www.ncbi.nlm.nih.gov/bioproject/PRJEB3381.

7 https://www.ncbi.nlm.nih.gov/bioproject/?term=PRJNA200694.

8 http://doi.org/10.5281/zenodo.10419127.

9 http://doi.org/10.5281/zenodo.10419127.

10 http://doi.org/10.5281/zenodo.10419127.

11 http://doi.org/10.5281/zenodo.10419127.

12 http://doi.org/10.5281/zenodo.10418867.
